# First steps matter: gait initiation reveals emotion-driven motor compensation in Parkinson's disease

**DOI:** 10.1093/braincomms/fcaf384

**Published:** 2025-10-06

**Authors:** Alessandro Botta, Tiziana Lencioni, Sara Terranova, Martina Putzolu, Gaia Bonassi, Ilaria Carpinella, Carola Cosentino, Francesca Lucchetti, Maurizio Ferrarin, Veronica Romano, Susanna Mezzarobba, Elisa Ravizzotti, Giovanna Lagravinese, Elisa Pelosin, Laura Avanzino

**Affiliations:** IRCCS Ospedale Policlinico San Martino, Genoa 16132, Italy; IRCCS Fondazione Don Carlo Gnocchi Onlus, Milan 20148, Italy; Department of Experimental Medicine, Section of Human Physiology, University of Genoa, Genoa 16132, Italy; IRCCS Ospedale Policlinico San Martino, Genoa 16132, Italy; Department of Experimental Medicine, Section of Human Physiology, University of Genoa, Genoa 16132, Italy; IRCCS Ospedale Policlinico San Martino, Genoa 16132, Italy; Department of Neuroscience, Rehabilitation, Ophthalmology, Genetics and Maternal Child Health, University of Genoa, Genoa 16132, Italy; IRCCS Fondazione Don Carlo Gnocchi Onlus, Milan 20148, Italy; Department of Neuroscience, Rehabilitation, Ophthalmology, Genetics and Maternal Child Health, University of Genoa, Genoa 16132, Italy; IRCCS Fondazione Don Carlo Gnocchi Onlus, Milan 20148, Italy; IRCCS Fondazione Don Carlo Gnocchi Onlus, Milan 20148, Italy; Department of Neuroscience, Rehabilitation, Ophthalmology, Genetics and Maternal Child Health, University of Genoa, Genoa 16132, Italy; IRCCS Ospedale Policlinico San Martino, Genoa 16132, Italy; Department of Neuroscience, Rehabilitation, Ophthalmology, Genetics and Maternal Child Health, University of Genoa, Genoa 16132, Italy; Department of Neuroscience, Rehabilitation, Ophthalmology, Genetics and Maternal Child Health, University of Genoa, Genoa 16132, Italy; IRCCS Ospedale Policlinico San Martino, Genoa 16132, Italy; IRCCS Ospedale Policlinico San Martino, Genoa 16132, Italy; Department of Neuroscience, Rehabilitation, Ophthalmology, Genetics and Maternal Child Health, University of Genoa, Genoa 16132, Italy; IRCCS Ospedale Policlinico San Martino, Genoa 16132, Italy; Department of Experimental Medicine, Section of Human Physiology, University of Genoa, Genoa 16132, Italy

**Keywords:** emotion, gait initiation, Parkinson’s disease, emotional facial expressions

## Abstract

Parkinson’s disease (PD) disrupts the intricate cognitive, emotional and sensorimotor circuits required for movement. In a recent study, we observed that fear-related embodied stimuli could enhance motor responses in early stage persons with PD (PwPD), potentially by activating neural compensatory mechanisms. In this observational study, we implemented a sensor-based ‘Go/No-go’ gait initiation task involving a response to emotional facial expressions—fear, happiness and neutral facial expressions—to elucidate whether and how emotional cues may drive compensatory mechanisms for motor performance in middle-stage PD. Furthermore, we investigated whether these mechanisms differ between PwPD exhibiting tremor-dominant (TD) or postural instability and gait disorders (PIGD) motor subtypes. By comparing gait initiation parameters between PD participants and age-matched healthy controls, we found that emotional stimuli reduced the duration of anticipatory postural adjustments and the step execution time across both groups, indicating a robust motor advantage. Specifically, happiness elicited a more pronounced advantage than fear, though PwPD displayed a diminished benefit relative to controls. Intriguingly, individuals with the PIGD subtype showed a weaker motor advantage for ‘happiness’ than those with the TD subtype, suggesting subtype-specific differences, possibly reflecting different underlying neural circuitry. Collectively, our findings reveal that while emotional cues generally facilitate gait initiation, fear and happiness exhibit distinct modulatory effects in PD. Compared to our earlier findings, the fear-related motor benefit appears to decline with disease progression, while the happiness-related advantage varies considerably across PD subtypes. These insights highlight the nuanced interplay between emotion and motor control in PD and suggest the use of a cognitive-emotional-sensorimotor integration task as a sensitive predictor for differentiating PD motor subtypes.

## Teaser

Botta *et al*. report that emotional cues, particularly happiness, facilitate gait initiation in middle-stage Parkinson’s disease (PD) and healthy controls. This advantage is diminished in persons with PD and differs between tremor-dominant and postural instability/gait disorder subtypes, suggesting subtype-specific compensatory mechanisms and a potential role for cognitive-emotional-sensorimotor tasks in differentiating PD motor subtypes.

## Graphical Abstract

**Graphical Abstract:**
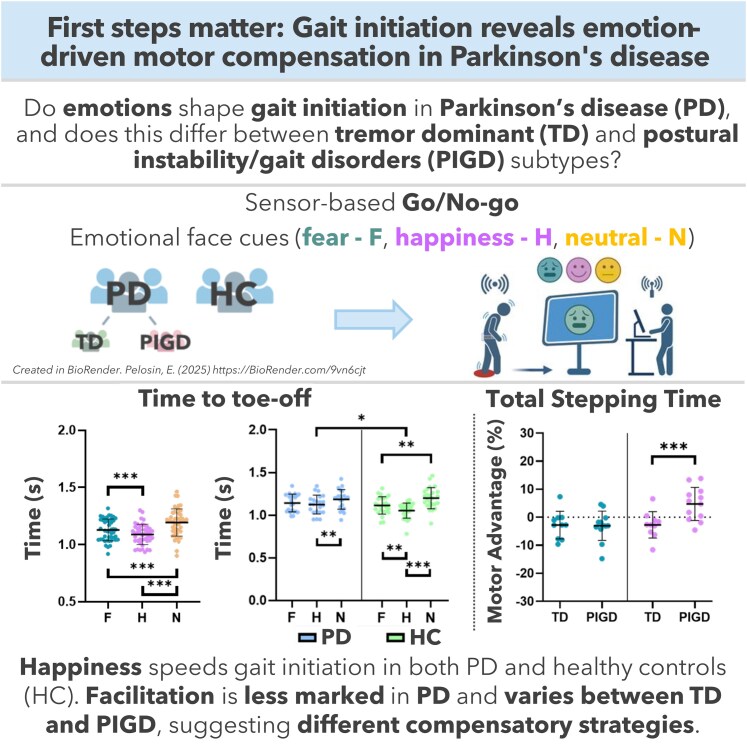

## Introduction

Motor behaviour is our means of interacting with the environment and involves a complex integration of cognitive, emotional and sensorimotor processes. Growing evidence suggests that emotions influence motor performance, modulating response times^1-3^ and whole-body movements.^4-6^ For instance, emotional stimuli like positive facial expressions and emotional body language can shorten motor response times, supporting the idea that emotions act as action dispositions for approach or avoidance behaviours.^1,2,7,8^ The integration of emotion and movement involves cortical and subcortical structures, including the sensorimotor cortex and basal ganglia.^9^ The basal ganglia integrate cognitive functions (such as attention and working memory) and emotional functions through segregated yet interconnected cortico-subcortical circuits, ensuring proper sensorimotor control.^10,11^

In Parkinson’s disease (PD), a neurodegenerative disorder characterized by the loss of dopaminergic neurons of the substantia nigra pars compacta, the integration of cognitive and sensorimotor processes is disrupted, altering the capacity to process information and control movement planning and execution concurrently.^11^ Interestingly, the limbic circuit appears relatively spared in PD,^11^ potentially compensating for motor deficits through emotional processing.^12^ Indeed, in PD, it has been described for many years the ‘kinesia paradoxa’ phenomenon, defined as ‘the sudden transient ability of a patient with PD to perform a task he or she was previously unable to perform’, as under emotional distress.^12^ Activation of limbic circuits (fed by the mesolimbic dopamine system and the limbic cortex) could automatically ‘energize’ the emotional locomotor system, as is indeed observed in kinesia paradoxa. The hypothesis of a compensatory role of the limbic circuit in PD aligns with the presence of a ‘third motor system’ proposed by Holstege in 1989, which is thought to control emotional expression and activate compensatory circuits under specific contextual or pathological conditions.^12,13^ Consistent with this idea, in a recent study^14^ we investigated upper limb motor response in a forced two-choice emotional discrimination task in 25 persons with PD (PwPD) in the early stage of the disease and 25 healthy, age-matched controls. Our findings indicate that negative emotional stimuli (i.e. fearful emotional body language and facial expressions) can hasten motor responses in early stage PD, strengthening the role of emotional pathways in compensatory mechanisms.^12,15^

Compensatory mechanisms are particularly relevant in PD due to their relationship with disease progression and clinical heterogeneity.^16^ Notably, the onset of motor symptoms of PD typically emerges only after significant nigrostriatal degeneration, suggesting that the disease manifests when the extent of striatal dopamine depletion exceeds the capacity of adaptive or compensatory mechanisms.^17^ Furthermore, when the motor aspects become manifest, interindividual differences in clinical severity emerge since the early stage of the disease and contribute to the heterogeneity in the severity of symptom progression, and hence in the prognosis of the disease.^16^

The specific weight of nigrostriatal degeneration and of compensatory mechanisms in influencing clinical heterogeneity and prognosis is currently under investigation: recently, Johannson *et al*.^18^ showed with a multimodal approach including clinical, behavioural and neuroimaging investigations, that cortical parieto-premotor compensation, rather than basal ganglia dysfunction, shapes interindividual variability in symptom severity in a large number of PwPD. So far, there have been different attempts to subtype PwPD on the basis of clinical manifestations (motor and non-motor symptoms) and progression, taking into account also neuroimaging and molecular markers.^16^

One approach to characterize PwPD involves classifying patients according to motor-predominant subtypes, namely tremor-dominant (TD) and postural instability and gait disorder (PIGD).^19,20^ These PD subtypes are not merely identifiable as stages of disease progression, but rather reflect distinct pathophysiological processes, which in turn reverberate in a more severe clinical course in the PIGD subtypes in terms of gait,^21,22^ cognitive^23,24^ and affective^25,26^ impairments compared to TD. Particularly, PIGD persons show more severe gait disturbances in terms of reduced gait speed, decreased stride length, increased stride variability, and impaired adaptive walking behaviours,^21,22^ as well as more frequent affective and depressive disorders compared to TD persons.^27^ Furthermore, from a neurophysiological point of view, recent imaging studies have explored differences between PD motor subtypes^18,28^ showing that TD persons show more localized alterations in the cerebello-thalamo-cortical pathway,^29,30^ which is associated with the onset of tremor. On the other hand, the PIGD motor subtype has shown a more aggressive neurodegenerative trajectory with a widespread disruption of both the dopaminergic (especially in the striato-thalamo-cortical network)^29^ and non-dopaminergic circuits (with higher degeneration at the level of the pedunculopontine nucleus and the basal forebrain).^31,32^

In the context of emotional pathways, Wang and colleagues,^33^ studying the resting-state functional connectivity of the subthalamic nucleus (STN), observed that PIGD persons have significantly lower STN-anterior cingulate cortex functional connectivity compared to TD persons, suggesting a reduced efficiency of the limbic pathway in the PIGD motor subtype.

Given the above-mentioned variability in disease progression and differences in clinical phenotype and underlying neurodegenerative trajectories between PIGD and TD, it appears relevant to explore how compensatory mechanisms involving emotional processing in PD evolve as the disease progresses and whether they differ between motor subtypes.

To address this gap, we first aimed to explore whether a gait initiation task could provide insights into the role of emotions in driving compensatory mechanisms of motor performance in middle-stage PD. For the purposes of this study, we defined mid-stage PD as a disease duration of 5 to 8 years, based on previous studies.^34^

We implemented a ‘Go/No-go’ gait initiation task, which required participants to respond to emotional facial expressions. The ‘Go/No-go’ task has been shown to involve basal ganglia,^35^ especially in selecting appropriate behaviours such as motor execution or response inhibition. This made it plausible to expect greater cognitive demand and potentially reduced performance in PwPD.^36^ We hypothesized that emotional stimuli would improve motor performance in PwPD by engaging compensatory neural pathways. However, unlike the effective compensatory mechanisms observed in early-stage PD,^14^ we expected a decline in efficiency in mid-stage PD.

Additionally, considering the more severe disease progression observed in PIGD compared to TD persons, we sought to determine whether step parameters in response to emotional stimuli could differentiate between middle-stage PwPD with specific motor subtypes. We hypothesized that the engagement of the emotional compensatory pathway would be less efficient in PIGD than in TD persons, contributing to the more severe clinical subtype observed in the latter.

## Materials and methods

### Study design

We designed an observational, within-subjects study, where all participants had to complete three randomly selected sessions of a sensor-based gait initiation emotional ‘Go/No-go’ task.

### Participants

A total of 30 PwPD (14 females; mean age ± SD: 68.64 ± 5.74 years old; H&Y: 2.1 ± 0.3) and 30 HC (15 females; mean age ± SD: 67.60 ± 5.73 years old) were recruited in the study. In a previous study, the IMU-based parameters for anticipatory postural adjustments (APA) used here demonstrated a large mean effect size (Cohen’s *d* = 0.83) when comparing PwPD to HC.^37^ Accordingly, with *α* = 0.05 and 1 − *β* = 0.80, G*Power 3.1 indicated that a minimum of 19 participants per group would be required. To account for potential technical issues or adherence-related challenges, we increased the sample size to 30 participants per group.

PwPD were recruited from the Centre for Parkinson's Disease at IRCCS San Martino of Genoa (Italy). Each participant provided written informed consent before inclusion in the study. The experimental protocol received approval from the regional ethics committee (CET Liguria; protocol number 662/2022) and was conducted following the principles outlined in the Declaration of Helsinki concerning experiments involving human participants.

### Eligibility criteria

All participants had to fulfil the following inclusion criteria: (i) age between 55 and 80 years old; (ii) Mini-Mental State Examination (MMSE) score > 24; (iii) clinical history not suggestive of neurological disease (other than PD for PwPD group); (iv) clinical history not suggestive for visual or orthopaedic impairments that could hinder task performance; (v) no history for psychiatric disorders. Additional criteria for PwPD were: (i) PD diagnosis (MDS criteria, 2015); (ii) disease duration: between 5 and 8 years; (iii) Hoehn and Yahr stage^38^ between 2 and 3; (iv) absence of deep brain stimulation implant.

All measures (i.e. clinical, neuropsychological and experimental) were performed in the morning (i.e. 9:00 a.m.). All PwPD were under standard dopaminergic therapy and in the ‘ON’ phase.^39^ Last therapy intake was monitored before the clinical evaluation; all PwPD reported administration within 90 min preceding the evaluation.

### Clinical and neuropsychological assessment

All PD participants underwent a clinical and neuropsychological assessment, performed by a neurologist and neuropsychologist specialized in movement disorders. Motor symptoms of PD were evaluated with the Italian version of the Movement Disorder Society-Unified Parkinson Disease Rating Scale, part III (MDS-UPDRS III), while the severity of freezing of gait (FOG) was assessed with the New Freezing of Gait Questionnaire (N-FOGQ).^40^ PD participants were categorized into two subgroups concerning their prevalent motor subtype: TD and PIGD subgroups^19^ and subsequently validated for the new MDS-UPDRS clinical scale.^41^ Non-motor symptoms were assessed via the MDS-UPDRS part II and the MDS-Non-motor Symptoms Scale (MDS-NMSS).^42^ Cognitive aspects were assessed using the PD-Cognitive Rating Scale (PD-CRS).^43^ Affective symptoms, including anxiety and depression, were evaluated via the Hamilton Anxiety Scale (HAM-A) and the Beck’s Depression Inventory II (BD-II), respectively.

### Task and experimental procedure

**Figure 1:**
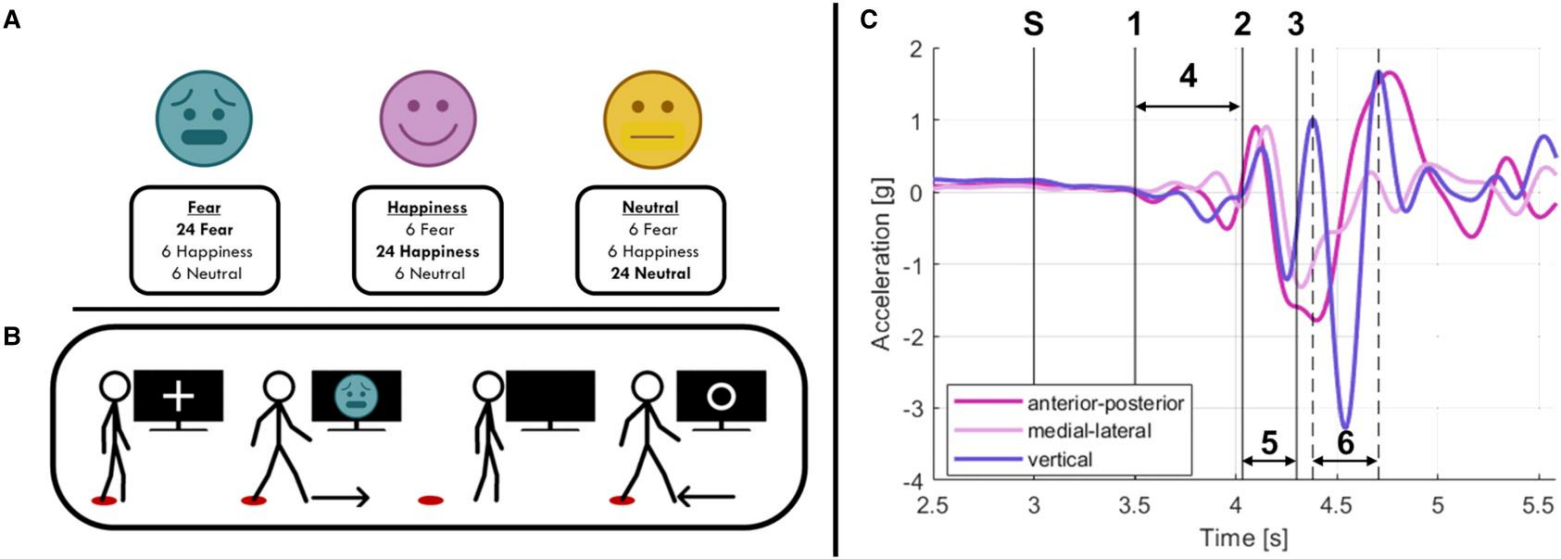
**Experimental protocol and gait parameters.** (**A**) Visual stimulus allocation for every emotional condition in the Go/No-go task. Counts shown in bold represent the ‘Go’ stimuli (*N* = 24); numbers in regular type indicate the ‘No-Go’ stimuli allocated to the other conditions. (**B**) Example of the experimental protocol in the ‘fear’ condition (i.e. fearful facial expressions as ‘Go’ signal, happy and neutral facial expressions as ‘No-Go’ signal; see text). The participants had to wait for the fearful facial expression to appear on the screen while observing a fixation cross, then step forward in response to the visual stimuli, and finally step back to the initial position as soon as the white circle appeared on the screen. A circle on the floor marked the start and finish positions. The experimental design was the same for all conditions (i.e. ‘fear’, ‘happiness’, and ‘neutral’). (**C**) Acceleration signals during gait initiation (example from one healthy participant): the first vertical line (*S* = Stimulus onset) represents the onset of the visual stimulus (i.e. ‘Go’ or ‘No-Go’ signal); (i) APA onset; (ii) beginning of the toe-off phase of the leading foot; (iii) heel trike of the leading foot; (iv) APA duration; (V) leading foot (step duration); (vi) trailing foot (step duration). Facial emotional stimuli were taken from Ekman’s Facial Action Coding System (FACS) test.^44^  **C** has been readapted from Lencioni *et al*.,^37^ with the permission and contribution of the copyright holder.

The experimental procedure is shown in Fig. 1A and B. Participants had to perform a single session of an emotional ‘Go/No-go’ task with three conditions: ‘fear’ (i.e. fearful facial expressions as ‘Go’ signal, happy and neutral facial expressions as ‘No-go’ signal), ‘happiness’ (i.e. happy facial expressions as ‘Go’ signal, fearful and neutral facial expressions as ‘No-go’ signal) and ‘neutral’(i.e. neutral facial expressions as ‘Go’ signal, fearful and happy facial expressions as ‘No-go’ signal). Participants had to wait for the ‘Go’ facial expressions to appear on the screen, then step forward as fast as possible in response to the visual stimulus, and finally step back to the starting position. The start/finish position was indicated by a red circle on the floor. At the appearance of the ‘No-go’ facial expression, participants were asked to withhold the motor response. The task was programmed using E-Prime 3.0 software (Psychology Software Tools, Pittsburgh, PA, USA) and synchronized with the inertial sensor’s acquisition software to record the gait data.

Emotional stimuli were taken from Ekman’s Facial Action Coding System (FACS) test.^44^ Specifically, the selected visual stimuli were drawn from a previous study conducted by our group on PwPD, in which they were validated for valence, arousal, and recognition accuracy. The stimuli showed an overall correct emotion recognition rate exceeding 97% for both fear and happiness, with no significant differences in valence or arousal ratings between PwPD and HC.^14^ A total of 20 pictures were selected for each of the three experimental conditions and randomly presented to the participants in the three ‘Go/No-go’ tasks. Each visual stimulus lasted for 500 ms, preceded by a fixation cross lasting for 3 s, and followed by a white circle lasting 13 s during which participants had to go back to the starting position. Visual stimuli were presented on a 55-inch LED TV positioned 3.5 m in front of the participants. Participants were instructed to stand still and received different instructions depending on the experimental condition: (i) take a step forward with the right leg, then complete the movement by bringing their left leg forward to a closed stance with aligned feet, as fast as possible when they recognized the ‘Go’ stimulus and then return to the initial position; and (ii) to remain still in response to the ‘No-go’ stimuli.

After a brief explanation of the experimental procedure, participants underwent a familiarization phase consisting of five trials to ensure they understood the task. Only the accelerometric data from the stimulus presentation to the alignment of the feet, recorded from a sensor placed on the lower back, were analysed.

The ratio between the ‘Go’ and the ‘No-go’ trials was 2:1, meaning that there were 24 ‘Go’ and 12 ‘No-go’ stimuli (for details, see Fig. 1A). A 5-min break was provided between conditions to mitigate fatigue.

### Data recording and parameters

The first step parameters were recorded at 128 Hz via 1 inertial sensor (Opals, by APDM Inc., Portland, Oregon, USA) placed at the lumbar level (i.e. L5 level). The recorded data were stored for offline analysis using a previously validated MATLAB (Mathworks Inc. Natick, Massachusetts, USA) script (for details, see Lencioni *et al*.^37^).

In the terminology of gait analysis, gait initiation refers to the phase between quiet standing and steady-state locomotion. The completion of the first step is usually considered the endpoint of the gait initiation phase. It can be further divided into a movement preparation phase and a movement execution phase. For the identification of the parameters related to the two phases, three key events during gait initiation were identified from the accelerometer data: APA onset, toe-off and heel strike related to the leading leg, according to the algorithm by Gazit *et al*.^45^ Subsequently, the two consecutive acceleration peaks, occurring after the identification of the leading leg's heel strike, were detected and used to determine the step timing of the trailing leg (see Gazit *et al*.^45^).

After this first framework, the following outcome measures were calculated for each phase: APA onset and APA duration for the movement preparation and the total stepping time for the execution period, estimated as the sum of the stepping times of the leading and trailing legs.

The definition of these gait parameters is described in Table 1, based on the acceleration profiles recorded by the inertial sensors shown in Fig. 1C.

**Table 1:** Gait parameters definition

Parameter	Definition
APA onset	Time-to-APA: time from the ‘GO’ signal (i.e. ‘S’ in Fig. 1C) to the beginning of the APA displacement (i.e. ‘1’ in Fig. 1C).
APA duration	Time from the beginning of the APA event (i.e. APA onset) to the end of the APA waveform (i.e. ‘4’ in Fig. 1C)
Time to toe-off	Time from the ‘GO’ signal (i.e. ‘S’ in Fig. 1C) to the APA end, calculated as the toe-off event of the leading leg (i.e. ‘2’ in Fig. 1C)
Time to heel strike	Time from the ‘GO’ signal (i.e. ‘S’ in Fig. 1C) to the heel strike of the leading leg (i.e. ‘3’ in Fig. 1C)
Total stepping time	Total time to complete the motor task required in the experiment, defined as the sum of ‘Leading foot step duration’ and ‘Trailing foot step duration’. ‘Leading foot step duration’ = Time to complete the first step with the leading foot, calculated as ‘Time to heel strike’—‘Time to toe-off’ (i.e. ‘5’ in Fig. 1C). ‘Trailing foot step duration’ = Time to complete the first step with the trailing foot, estimated as the time difference between the two consecutive vertical acceleration peaks after the heel strike of the leading foot (i.e. ‘6’ in Fig. 1C); for details see Gazit *et al*.^36^

The table shows the definition of the temporal gait parameters used as outcome measures in the experiment. In the definition, there is also a reference to the events shown in Fig. 1C.

APA, anticipatory postural adjustments; S, stimulus onset.

### Data and statistical analysis

The distribution’s normality of the data was assessed through the Shapiro–Wilk test, and sphericity through Mauchly’s test. Clinical and neuropsychological data for PwPD were analysed via a non-parametric independent-samples Mann–Whitney test to explore clinical differences between the two motor subtypes (i.e. TD and PIGD).

Accuracy was computed as the percentage of errors committed in the ‘GO’ trials. We considered a trial as an error when a participant did not respond to the correct ‘GO’ stimulus or when the first step was not completed. Accuracy data were first rank-transformed and then analysed via an aligned rank transformed analysis of variance (ART-ANOVA) with ‘Group’ (PwPD and HC) as between between-subjects factor and ‘Emotion’ (fear, happiness and neutral) as within-subject factor.

Temporal gait parameters (i.e. ‘APA onset’, ‘APA duration’, ‘Time to toe-off’, ‘Time to heel strike’ and ‘Total stepping time’) were analysed via a repeated measure analysis of variance (RM-ANOVA) with ‘Group’ (PwPD and HC) as between-subject factor and ‘Emotion’ (fear, happiness and neutral) as within-subject factor. The analysis was run separately for each parameter.

To better understand the effect of emotional stimuli on the ‘Go/No-go’ gait initiation task, we computed the motor advantage for each temporal gait parameter as follows:


(1) $$\begin{array}{r} \mathrm{Motor}\;\mathrm{advantage}=\frac{\left(\mathrm{Emotional}\;\mathrm{data}-\mathrm{Neutral}\;\mathrm{data}\right)}{\mathrm{Neutral}\;\mathrm{data}}\times 100 \end{array}$$

A positive index value indicates an increased temporal parameter, hence a lack of facilitation, in the emotional condition compared to a neutral one, whereas a negative index value indicates a shorter temporal parameter, hence a facilitatory effect of emotions on gait initiation. Motor advantage data were analysed via an RM-ANOVA with ‘Group’ (PwPD and HC) as between-subject factor and ‘Emotion’ (fear and happiness) as within-subject factor. The analysis was run separately for each of the step parameters. Furthermore, motor advantage data were analysed via an independent *t*-test to explore whether the motor subtype (TD versus PIGD) of the disease could affect cognitive–emotional processing. Bonferroni correction for multiple comparisons was implemented in *post hoc* analysis.

Post-hoc sensitivity analysis was run for both gait parameters and motor advantage data for each of the effects included in the statistical analysis.

Spearman’s rank correlation coefficient method was used to investigate correlations between step parameters motor advantage, and clinical and neuropsychological data. Results were corrected for multiple comparisons following the method proposed by Curtin and Schulz.^46^ Lastly, a simple binary logistic regression analysis, with ‘Subtype’ (i.e. TD = 0 and PIGD = 1) as the dependent variable and ‘Motor advantage’ as the independent variable, was performed on motor advantage data that showed significant differences between TD and PIGD subtypes, to explore whether the performance on the emotional ‘Go/No-go’ task could predict the subtype of PwPD. An S-shaped (sigmoidal) logistic curve and a Receiver Operating Characteristic (ROC) curve were generated, the first showing the relationship between predictor values (i.e. ‘Motor advantage’) and the estimated outcome (i.e. ‘Subtype’) probability, and the second to quantify the overall ability of the task to discriminate between the two motor subtypes.

Statistical analysis was run on the R-based software BlueSky Statistics^©^ v.10.3.4 (Chicago, USA).

## Results

### Participants

Data from 22 PwPD (10 TD and 12 PIGD) and 25 HC entered the analysis. Participants were excluded when the accuracy of one or more conditions was lower than 50% (i.e. more than 12 errors in the ‘Go’ condition), resulting in the exclusion of 5 PwPD and 5 HC. Furthermore, 3 PwPD were excluded from the analysis because they were unable to complete the experimental session for fatigue. Raw data were corrected for outliers, removing values above and below the mean value plus/minus two standard deviations. The two groups were matched for age (*P* = 0.545) and sex (*P* = 0.111). Participants’ characteristics are reported in Table 2. The Mann–Whitney test run on clinical and neuropsychological data revealed no significant differences in the comparison between the two PD subtypes (i.e. TD and PIGD), except for the Levodopa equivalent daily dose (*P* = 0.030) and for the MDS-UPDRS part II (*P* = 0.047), which resulted to be higher for the PIGD subtype. Results are reported in Table 2.

**Table 2:** Demographic, clinical and neuropsychological characteristics

Gender	PwPD	HC	*P*-value	TD	PIGD	*P*-value
	(14M. 8F)	(10M. 15F)	*P* = 0.11	(5M. 5F)	(9M. 3F)	
	Mean ± SD	Mean ± SD				
Age (years)	68.64 ± 5.74	67.60 ± 5.7	*P* = 0.55	67.40 ± 5.90	69.67 ± 5.39	*P* = 0.38
Hoehn & Yahr (stage)	2.1 ± 0.3			2.1 ± 0.3	2.2 ± 0.4	*P* = 0.82
LEDD (mg/die)	602.27 ± 300.62			439.20 ± 229.89	738.17 ± 284.63	***P*** **=** 0.030*
Disease duration	6.50 ± 1.07			6.20 ± 1.25	6.75 ± 0.83	*P* = 0.25
MDS-UPDRS part II	7.86 ± 8.19			4.10 ± 6.20	11.27 ± 8.28	***P*** **=** 0.047*
MDS-UPDRS part III	28.96 ± 12.62			32.87 ± 11.27	25.67 ± 12.75	*P* = 0.20
NFOG-Q	5.55 ± 8.60			5.10 ± 2.36	5.91 ± 1.65	*P* = 0.54
MDS-NMSS	88.42 ± 63.35			61.78 ± 45.67	112.64 ± 67.33	*P* = 0.08
MMSE	27.60 ± 2.08			27.01 ± 2.36	28.08 ± 1.65	*P* = 0.31
PD-CRS TOT	94.06 ± 11.58			90.37 ± 12.07	93.60 ± 12.55	*P* = 0.09
PD-CRS subcortical	27.032 ± 2.52			62.21 ± 12.01	66.10 ± 11.59	*P* = 0.44
PD-CRS cortical	64.16 ± 11.96			27.35 ± 1.50	26.71 ± 2.77	*P* = 0.99
HAM-A	10.21 ± 5.61			9.78 ± 3.61	10.6 ± 6.9	*P* = 0.78
BDI-II	14.95 ± 8.56			15.44 ± 9.01	14.50 ± 8.11	*P* = 0.78

The table presents the characteristics of the participants and the results of the statistical analysis comparing the groups.

Legend: PwPD, Persons with Parkinson’s Disease; HC, Healthy Control; TD, Tremor Dominant; PIGD, Posture Instability and Gait Disorders; LEDD, Levodopa Equivalent daily dose; MDS-UPDRS part II-III, Movement Disorder Society Unified Parkinson’s Disease Rating Scale; New Freezing of Gait questionnaire; Movement Disorder Society Non-Motor Symptoms Scale; Mini-mental Scale Evaluation; PD-CRS, Parkinson’s Disease Cognitive Rating Scale; HAM-A, Hamilton Anxiety Rating Scale; and Beck’s Depression Inventory-II.

Significant statistical differences are reported in bold. *P-value below the significance threshold of 0.05.

### Accuracy

Accuracy was high for both groups. PwPD showed an accuracy of 87.1% in the ‘Fear GO’ trial, 87.3% in the ‘Happiness GO’ trial and 90.3% in the ‘Neutral GO’ trial. Similarly, HCs have shown an accuracy of 90.0% in the ‘Fear Go’ trial, 89.7% in the ‘Happiness GO’ trial and 87.3% in the ‘Neutral GO’ trial. ART-ANOVA results showed no significant ‘Group’, ‘Emotion’ or interaction effects (all *F* < 1 and *P* > 0.05).

### Gait initiation

**Figure 2:**
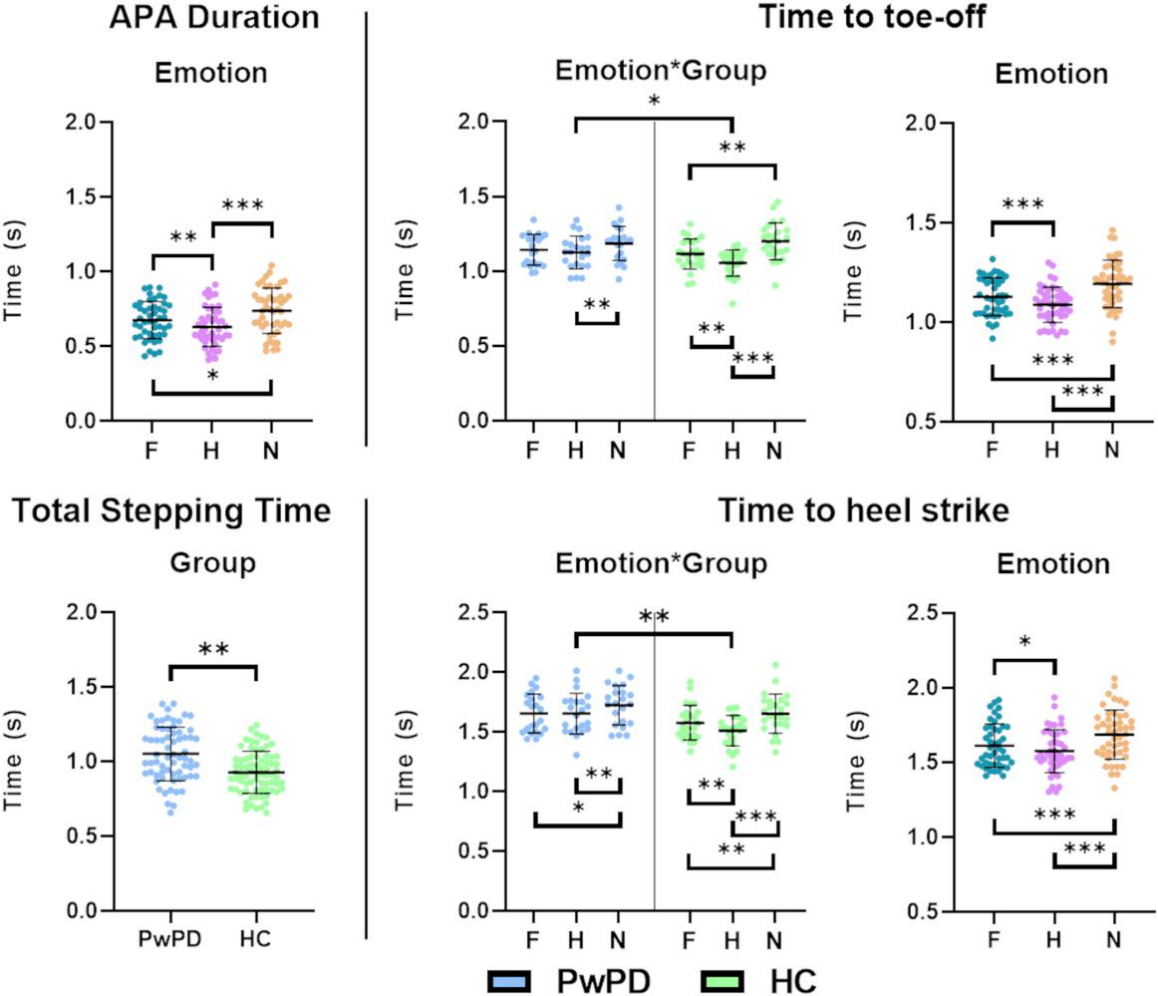
**Results of statistical analysis on raw gait data.** The figures display only the gait parameters for which a significant difference was found in the RM-ANOVA. For ‘APA Duration’, the figure shows the main effect ‘Emotion’, while for ‘Total stepping time’, it shows the main effect ‘Group’. For ‘Time to toe-off’ and ‘Time to heel strike’, it has been reported the interaction effect ‘Emotion * Group’ and the main effect ‘Emotion’. Data are reported as the mean (thick black line) ± standard deviation. Exact statistics are reported in the results. *F* = Fear; H = Happiness; *N* = Neutral; PwPD = Persons with Parkinson’s disease (*N* = 22); HC = Healthy controls (*N* = 25); *P < 0.05; **P < 0.01; ***P < 0.001.

Gait initiation parameters are shown in Fig. 2.

We ran a *post hoc* sensitivity analysis using G*Power 3.1 for each of the factors implemented in the statistical analysis, based on our design (*N* = 47, *α* = 0.05, 1 − *β* = 0.80). Results revealed that the minimum detectable effect sizes were as follows: (i) *f* = 0.34 for the between-subjects factor ‘Group’; (ii) *f* = 0.27 for the within-subjects factor ‘Emotion’; (iii) *f* = 0.43 for the interaction effect ‘Group*Emotion’. The observed effect for each gait parameter was compared to these thresholds (see below).

The RM-ANOVA for ‘APA onset’ revealed no significant main effects for ‘Emotion’ and ‘Group’, nor any interaction effects (all *F* < 1 and *P* > 0.05).

For ‘APA duration’, a significant effect for ‘Emotion’ was observed (*F*(2,90) = 19.001; *P* < 0.001; *pη*^2^ = 0.297). Post-hoc analysis revealed that ‘APA duration’ was longer for neutral conditions compared to both fear (*P* = 0.017) and happiness (*P* < 0.001), and for fear compared to happiness (*P* < 0.01). No significant effects were observed for ‘Group’ (*F*(1,45) = 3.402; *P* = 0.072; *pη*^2^ = 0.070) and for the interaction ‘Group*Emotion’ (*F*(2,90) = 0.882; *P* = 0.417; *pη*^2^ = 0.019). Sensitivity analysis showed an above-threshold, large effect size for ‘Emotion’ (*f* = 0.65).^47^

For ‘Time to toe-off’, statistical analysis showed no significant differences between groups (*F*(1,45) = 1.027; *P* = 0.316; *pη*^2^ = 0.022). However, significant effects were found for ‘Emotion’ (*F*(2,90) = 31.157; *P* < 0.001; *pη*^2^ = 0.409) and for ‘Group*Emotion’ interaction (*F*(2,90) = 4.155; *P* = 0.026; *pη*^2^ = 0.085). Post-hoc analysis of the ‘Emotion’ factor revealed faster step initiation for the emotional conditions compared to neutral (‘fear’ versus ‘neutral’: *P* < 0.001; ‘happiness’ versus ‘neutral’: *P* < 0.001) and in the ‘happiness’ condition compared to ‘fear’ (*P* < 0.001). Post-hoc analysis of the interaction showed that ‘Time to toe-off’ was shorter in HC in the ‘happiness’ and ‘fear’ conditions compared to ‘neutral’ (*P* < 0.01) and in the ‘happiness’ condition relative to ‘fear’ (*P* < 0.01). In PwPD, ‘Time to toe-off’ was shorter only in the ‘happiness’ condition compared to ‘neutral’ (*P* < 0.01). Furthermore, HC had a shorter ‘Time to toe-off’ than PwPD in the ‘happiness’ condition (*P* = 0.028). Post-hoc sensitivity analysis showed a large effect size for ‘Emotion’ (*f* = 0.83), and a moderate-to-high effect size for the interaction effect ‘Group*Emotion’ (*f* = 0.31), although slightly below the computed threshold.

Similarly for ‘Time to heel strike’, statistical analysis showed a significant effect for ‘Emotion’ (*F*(2,90) = 26.083; *P* < 0.01; *pη*^2^ = 0.367), for the ‘Emotion*Group’ interaction (*F*(2,90) = 4.291; *P* = 0.019; *pη*^2^ = 0.087), and for ‘Group’ (*F*(1,45) = 5.018; *P* = 0.030; *pη*^2^ = 0.100). Post-hoc analysis for the ‘Group’ main effect revealed a longer time to heel-strike for PwPD compared to HC. Post-hoc analysis for ‘Emotion’ main effect showed a shorter ‘Time to heel strike’ for the emotional conditions compared to neutral (‘fear’ versus ‘neutral’: *P* < 0.001; ‘happiness’ versus ‘neutral’: *P* < 0.001) and in the ‘happiness’ condition compared to ‘fear’ (*P* = 0.020). Regarding the ‘Group*Emotion’ interaction, the *post hoc* analysis revealed that Time to heel-strike was shorter in HC in the ‘happiness’ and ‘fear’ conditions relative to ‘neutral’ (always *P* < 0.001) and in the ‘happiness’ condition compared to ‘fear’ (*P* < 0.01). In PwPD, ‘Time to heel-strike’ was shorter in the ‘happiness’ and in the ‘fear’ conditions compared to ‘neutral’ (*P* < 0.01 and *P* = 0.040, respectively), but not in the ‘happiness’ condition compared to ‘fear’ (*P* > 0.05). Furthermore, HC showed a shorter time to heel-strike than PwPD in the ‘happiness’ condition (*P* < 0.01). Sensitivity analysis showed a large effect size for ‘Emotion’ (*f* = 0.76), and a moderate-to-high effect size for the main effect ‘Group’ (*f* = 0.33) and the interaction effect ‘Group * Emotion’ (*f* = 0.31).

Finally, analysis of ‘Total stepping time’ revealed a significant effect for ‘Group’ (*F*(1,45) = 8.101; *P* < 0.01; *pη*^2^ = 0.153), where HC resulted in being faster compared to PwPD. Sensitivity analysis on the ‘Group’ effect showed an *f* = 0.43.

### Motor advantage data

**Figure 3:**
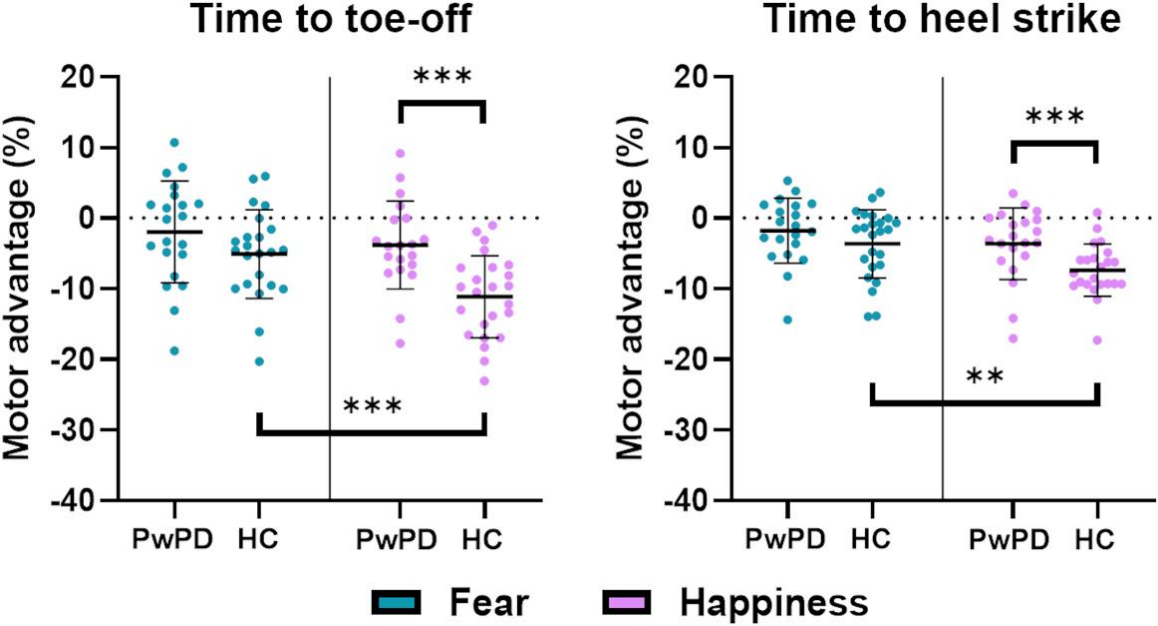
**Results of the statistical analysis on motor advantage data.** The figure shows the gait parameters that showed a significant ‘group * emotion’ interaction effect in the RM-ANOVA. A negative value of the motor advantage means that participants were facilitated in the emotional conditions compared to the non-emotional condition. Data are reported as the mean (thick black line) ± standard deviation. Exact statistics are reported in the results. *F* = Fear; H = Happiness; *N* = Neutral; PwPD = Persons with Parkinson’s disease (*N* = 22); HC = Healthy controls (*N* = 25); **P < 0.01; ***P < 0.001.

Motor advantage data and statistical analysis results are illustrated in Fig. 3. Statistical analyses on gain data for ‘fear’ and ‘happiness’ conditions were run separately for each of the first-step parameters. Based on our design (*N* = 47, *α* = 0.05, 1 − *β* = 0.80), *post hoc* sensitivity analysis results revealed that the minimum detectable effect sizes were as follows: (i) *f* = 0.36 for the between-subjects factor ‘Group’; (ii) *f* = 0.30 for the within-subjects factor ‘Emotion’; (iii) *f* = 0.42 for the interaction effect ‘Group * Emotion’. Each gait parameter's observed effects were individually compared to these thresholds.

Results for motor advantage of ‘APA onset’, of ‘APA duration’, and of ‘Total stepping time’ showed non-significant differences (all *F* < 1 and *P* > 0.05).

For ‘Time to toe-off’, significant results were retrieved for the main effects ‘Group’ (*F*(1,40) = 7.588; *P* < 0.01; *pη*^2^ = 0.159), where a higher advantage was observed for HC compared to PwPD, ‘Emotion’ (*F*(1,40) = 22.160; *P* < 0.01; *pη*^2^ = 0.356), where a higher advantage was retrieved for happy compared to fearful stimuli, and for the interaction ‘Group*Emotion’ (*F*(1,40) = 4.541; *P* = 0.039; *pη*^2^ = 0.102). As for the interaction effect, the *post hoc* analysis revealed a lower motor advantage for PwPD compared to HC in response to happy stimuli (*P* < 0.001), and a generally higher advantage for ‘happiness’ compared to ‘fear’ for HC (*P* < 0.001), but not for PwPD, where it was observable a trend (*P* = 0.080). All effect sizes resulted from the analysis were above the threshold computed in the sensitivity analysis (i.e. *f* = 0.43 for ‘Group’, *f* = 0.34 for ‘Emotion’, and *f* = 0.74 for ‘Group*Emotion’).

For ‘Time to heel strike’, no significant effect was observed for ‘Group’ (*F*(1,40) = 1.939; *P* = 0.171; *pη*^2^ = 0.041). However, significant results were found for ‘Emotion’ (*F*(1,40) = 9.944; *P* < 0.01; *pη*^2^ = 0.181), where an increased advantage was retrieved for ‘happiness’ compared to ‘fear’, and for Group * Emotion’ interaction (*F*(1,40) = 4.318; *P* = 0.043; *pη*^2^ = 0.088). Post-hoc analysis revealed a significantly higher advantage for ‘happiness’ compared to ‘fear’ for HC (*P* < 0.001), and a lower advantage for happy stimuli, for PwPD compared to HC (*P* = 0.024). Sensitivity analysis showed an adequate power for ‘Emotion’ (*f* = 0.47), and a slightly below threshold effect size for ‘Group * Emotion’ (*f* = 0.31).

Results of the correlation analysis, after the correction for multiple comparisons (i.e. corrected *P* ≤ 0.003), showed a positive correlation between ‘Total stepping time’ (*r* = 0.673; *P* = 0.001) and ‘Subtype’, indicating that a lesser motor advantage (i.e. more positive motor advantage value) is correlated with the PIGD motor subtype. Additionally, we found significant positive correlations between the ‘APA duration’ for both ‘fear’ (*r* = 0.497; *P* = 0.003) and ‘happiness’ (*r* = 0.666; *P* = 0.001) and the LEDD, showing that the higher the duration of the APA, the higher the LEDD intake of the PwPD. No significant correlations were found for the other variables.

### Motor advantage data: TD versus PIGD

**Figure 4:**
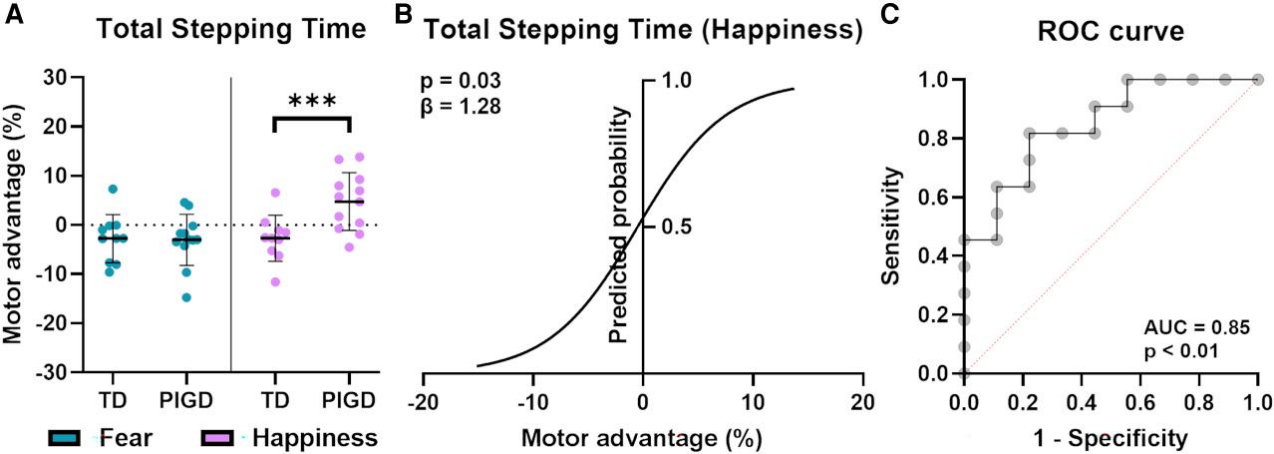
**Results of motor advantage data subgroup and binary logistic regression analyses.** (**A**) Results of motor advantage data subgroup analysis. The figure shows the results of the independent *t*-test performed considering the subgroups ‘TD’ (*N* = 10) and ‘PIGD’ (*N* = 12) of PwPD, for ‘fear’ and ‘happiness’. As observable, the only significant differences are retrievable in terms of a loss of motor advantage in the ‘happiness’ condition in the ‘PIGD’ motor subtype subgroup; (**B**) S-shaped logistic curve for ‘Total stepping time’ for the ‘happiness’ condition computed for all PwPD (*N* = 22). The curve shows the probability (y-axis) of being in the ‘PIGD’ subgroup versus motor advantage (*x*-axis). The lower the motor advantage (i.e. positive motor advantage value) in the ‘happiness’ condition, the higher the likelihood of identifying persons with a ‘PIGD’ subtype; (**C**) ROC curve for ‘Total stepping time’ for the ‘happiness’ condition (*N* = 22). The curve shows that the ‘Total stepping time’ motor advantage in the emotional ‘Go/No-go’ task can reliably distinguish between PIGD and TD subtypes in Parkinson’s disease, in the happiness condition. An AUC of 0.85 indicates a strong predictive ability. The dotted red line represents the ‘line of no discrimination’. Data are reported as the mean (thick black line) ± standard deviation. Exact statistics are reported in the results. ***P < 0.001; *β* = Odds ratio; AUC = Area under the ROC curve.

Significant differences between the two PD subtypes, TD and PIGD, were found only in the ‘happiness’ condition in ‘Total stepping time’. The independent *t*-test run on motor advantage data showed significant differences between TD and PIGD persons (*P* < 0.01), where PIGD showed no facilitation (see Fig. 4A). Post-hoc sensitivity analysis indicated a Cohen’s *d* of 1.26 (*α* = 0.05, and 1 − *β* = 0.80) as the minimum detectable effect size. We retrieved an actual Cohen’s *d* = 1.28, indicating a robust difference between the two subgroups. Logistic regression analysis was performed on motor advantage data for ‘Total stepping time’ in the ‘happiness’ condition (see Fig. 4B). Firstly, the Hosmer–Lemeshow test was conducted to assess the model’s goodness of fit, indicating an acceptable fit of the model to the observed data (*P* = 0.841). The logistic regression revealed a significant coefficient for ‘Total stepping time’ (*β* = 0.250) with an associated *Z* value of 2.106 and a *P*-value of 0.03. Results suggest that a worse performance in the integration of cognitive–emotional-sensorimotor information during positive emotional processing increases the likelihood of identifying a patient with a PIGD subtype. More specifically, the coefficient translates to an odds ratio (OR) of 1.28 (95% CI = [1.069, 1.754]), meaning that each unit of reduced motor advantage in the task increases the odds of falling in the PIGD subgroup by 28%. Moreover, the ROC curve for the model (see Fig. 4C demonstrated an area under the curve of 0.85; *P* < 0.01), suggesting that the motor advantage—reflected in the time required to execute the first step during a cognitive-emotional-sensorimotor task—is an excellent predictor of PD subtypes.^48^

## Discussion

The main aim of the present study was to explore whether a gait initiation task can be used to investigate the role of emotion in driving compensatory mechanisms of motor performance in PwPD in the middle stage of the disease and with different motor subtypes. To this aim, step temporal parameters were recorded during an emotional Go/No-go task, where participants were asked to step forward as fast as possible in response to emotional facial expressions depicting fear, happiness, or in response to neutral facial expressions.

The key findings are as follows:

- Emotional influence on gait initiation: In both PwPD and HC, gait initiation is influenced by emotional manipulation, with ‘APA duration’, ‘Time to toe-off’, and ‘Time to heel strike’ shorter in the emotional conditions in comparison to neutral, indicating a motor advantage in response to emotional stimuli.
- Enhanced motor advantage for ‘happiness’: In both PwPD and HC, the modulation of step temporal parameters in response to emotional stimuli was higher for ‘happiness’ stimuli compared to ‘fear’ stimuli, suggesting a greater motor advantage in response to positive stimuli.
- Group differences in motor advantage: In PwPD, motor advantage in response to ‘fear’ stimuli was not different in comparison to HC, whereas in response to ‘happiness’ stimuli, the motor advantage was smaller compared to HC.
- Subtype-specific motor response: In PwPD, in response to ‘happiness’ stimuli, the motor advantage was different depending on the motor subtype: in the PIGD subtype, it was smaller compared to the TD subgroup, and ‘Total stepping time’ motor advantage in response to ‘happiness’ stimuli was a valid predictor for PIGD persons.

### Emotional influence on gait initiation and enhanced motor advantage for happiness

Gait initiation is typically thought to be complete when the first stride is taken and can be divided into two periods: the preparation and the execution phases. ‘APA onset’ and ‘APA duration’ reflect the efficiency of the preparation phase, whereas ‘Time to toe-off’, ‘Time to heel strike’, and ‘Total stepping time’ represent the execution phase.

Concerning the effect of emotional stimuli on the ‘Go/No-go’ gait initiation task, we found faster movements in response to emotional stimuli (both ‘happiness’ and ‘fear’) compared to the non-emotional condition. These findings indicate a motor advantage associated with emotional stimuli. Furthermore, results also showed that the motor advantage was higher in response to ‘happiness’ than to ‘fear’. The effects of emotional processing on first-step parameters were found already in the preparatory phase of gait initiation (APA duration) and were present until the execution phase of the first step (i.e. ‘Time to toe-off’ and ‘Time to heel strike’), indicating that both the motor program and execution phases are accelerated in response to emotional stimuli. These findings are consistent with previous research indicating that the emotional valence of a stimulus modulates both the planning and execution of goal-directed movements.^4,6,49^ For instance, Naugle and co-workers^49^ demonstrated that highly arousing unpleasant emotional states accelerate the initial motor response, while pleasant emotional states facilitate the initiation of forward gait due to the approach-oriented directional salience of the movement. Similarly, studies on upper limb movements have shown that reaching and grasping tasks are more efficiently performed in response to pleasant stimuli.

The role of emotional stimuli during the preparation phase of a motor task can be explained by considering the role of emotions in sensory processing. Emotional processing facilitates sensory processing in the visual^50,51^ and somatosensory^14,52,53^ domains to prepare for action (i.e. avoidance or approach-related behaviours).

The larger motor advantage in response to ‘happiness’ as opposed to ‘fear’ stimuli might stem from the task requiring participants to step toward a positive stimulus, which aligns with innate approach behaviours.^54^ Conversely, stepping toward ‘fear’ stimuli goes against the innate avoidance behaviour, potentially inducing the ‘freezing’ phenomenon.^55,56^

### Group differences in motor advantage and subtype-specific motor response in PwPD

When comparing gait initiation performance between PwPD and HC subjects, several differences emerged, independent of the emotional context. Specifically, ‘Time to heel strike’ and ‘Total stepping time’ were significantly longer in PwPD in contrast to HC, indicating slower gait, primarily caused by bradykinesia and rigidity/hypertonia.^57,58^ Differently, ‘APA onset’ and ‘APA duration’ were similar between PwPD and HC. These results align with studies conducted on mild-to-moderate PD participants, treated with Levodopa, which showed general preservation of postural mechanisms during preparatory phases of gait initiation,^59^ especially in response to external cues.^60^

Regarding the effect of the specific emotional stimuli (i.e. ‘happiness’ and ‘fear’), both HC and PwPD groups demonstrated a motor advantage in the execution phase in response to emotional stimuli compared to neutral stimuli. However, while PwPD and HC showed a similar motor advantage in response to ‘fear’ stimuli, the motor advantage in response to ‘happiness’ was smaller in PwPD compared to HC.

Regarding the motor response to ‘fear’ stimuli, we recently assessed upper limb response times to emotional stimuli in early-stage PwPD.^14^ As already stated in the Introduction, our findings revealed that response times were faster when facing potential threats (i.e. ‘fear’ stimuli) compared to pleasant or non-emotional stimuli in both HC and PwPD, with a greater effect in PwPD versus HC. We hypothesized that fearful embodied stimuli might have triggered compensatory mechanisms in early PD, resulting in the activation of alternative motor pathways, such as the emotional basal ganglion module, compatible with the ‘kinesia paradoxa’ phenomenon.^10,15,61^

In this study, involving middle-stage PwPD and a more complex gait initiation task, we continued to find a motor advantage in response to ‘fear’ stimuli, which, however, was not larger in PwPD than HC. Importantly, in this as in our previous study, we also observed that PwPD were as accurate as healthy controls in recognizing facial emotional stimuli, excluding the potential impact of emotion recognition as a confounding factor.^14^

The differences in behavioural responses to ‘fear’ stimuli found in the two studies can be linked either to different methodological aspects or to different clinical characteristics of PwPD. Indeed, in the first study, we recorded upper limb response time when participants were asked to recognize the emotional content of pictures in a three-alternative forced choice task,^8^ whereas in this study, we adopted a more complex ‘Go/No-go’ whole body movement involving a gait initiation task. Furthermore, in our first study, we recruited persons in the early stage of the disease (i.e. disease onset within 5 years; Hoehn and Yahr stages 1–2). Here we recruited persons in the middle stage of the disease (i.e. disease duration between 5 and 8 years and Hoehn and Yahr stage between 2 and 3). Following our a priori hypothesis described in the Introduction, we can suggest that the putative compensatory mechanisms driven by ‘fear’ stimuli acting in the early stage of the disease deteriorate with disease progression. This result partially aligns with what we have shown in Lagravinese *et al*.,^5^ where worsened gait initiation in PwPD with freezing of gait was observed when participants were asked to step in response to negative ‘fear’ emotional stimuli. Following this line of reasoning, a possible loss of efficiency in compensatory mechanisms involving the emotional basal ganglion module could have been responsible for the worsening of motor symptoms, as in the case of FOG.

Related to the motor response to ‘happiness’ stimuli, it remains to explain why PwPD showed a reduced motor advantage in the execution phase of gait initiation compared to HC. Data in the literature suggest that this result is unlikely to be linked to deficits in facial emotion recognition, which, on the other hand, has been shown to arise in more severe phases of the disease, especially in PwPD with higher motor dysfunctions (i.e. H&Y ≥ 3, and UPDRS III > 35 points).^62,63^ Furthermore, as highlighted in a recent review by Argaud *et al*., ^63^ happy facial expressions are recognized more quickly and accurately than other emotions by both healthy subjects and PwPD, likely due to their most distinctive configuration, which features a very salient characteristic: the smile.^63^

Rather, the lower motor advantage can be due to the reduced ability of PwPD to hasten motor response when facing happy emotional stimuli. Notably, the response of PwPD to ‘happiness’ stimuli differed in PwPD with different motor subtypes. Indeed, in PIGD persons, the motor advantage in response to ‘happiness’ stimuli was smaller compared to TD and HC. The model’s ROC curve demonstrated a high predictive capacity of the motor advantage elicited by ‘happiness’ stimuli in differentiating PD subtypes. This finding can partly explain why our results are in contrast with what was observed by Naugle and co-workers, who showed, by using positive emotional scenes taken from the IAPS, that the exposure to pleasant images facilitated the momentum needed to move forward and reach single limb support during forward gait initiation, similarly for PwPD in the moderate stage of the disease and healthy controls.^6^ Indeed, Naugle and co-workers did not differentiate the gait behaviour in response to positive stimuli concerning different motor subtypes in PD.

To infer a possible explanation for our result is important to remember that the motor advantage in response to ‘happiness’ stimuli has been already described in healthy subjects for complex movements like gait initiation in response to pleasant stimuli or like reaching, grasping and bringing to body pleasant objects, supporting the behaviour-oriented nature of emotions that favour approach behaviours in response to emotion balanced positive stimuli.^54^ It can be speculated that one of the differences between PIGD and TD subtypes lies in their differential ability to translate a positive motivational state, such as ‘happiness’, into an appropriate physiological response, namely a facilitated motor reaction. This can be a consequence of differences in underlying neural circuitry between subtypes described in the literature.^64^ It has been suggested that there may be variations in the underlying brain circuitry between motor subtypes in PD, as evidenced by the negative relationship between the PIGD score and subthalamic–putamen connectivity and the tremor score and subthalamic–cerebellum connectivity.^33^ According to another study, voxel-based morphometry MRI investigations revealed more grey matter atrophy in the prefrontal cortex and globus pallidus in people with the PIGD subtype.^65^ Taking into consideration that the prefrontal cortex is highly connected to motor cortical areas and basal ganglia, and that it is a crucial node in the network responsible for translating emotion into action (for a review, see Braine *et al*.^66^), the differences in structure and connectivity of this area between PD motor subtypes could theoretically explain differences in emotion-related behaviour. However, exploring whether the reduced motor advantage in PIGD persons is linked to abnormal connectivity at a neural level was beyond the aim of this study and should be addressed in an ad hoc study. Related to why only the motor advantage in response to ‘happiness’ stimuli and not the one in response to ‘fear’ stimuli differed between PIGD and TD persons, we can only make conjectures. We can speculate that whereas ‘fear’ stimuli may activate compensatory mechanisms because of their evolutionary nature in TD and PIGD subtypes, the motor behaviour in response to ‘happiness’ stimuli may differentiate the motor subtype, likely because of differences in underlying neural circuitry.

### Limitations and future directions

Some limitations to this study must be considered. First, the results of the logistic regression should be handled with caution because of the reduced numerosity of the sample. Although the statistical results are significant, logistic regression requires a bigger sample size to propose a generalized and clinically valid result. In this context, expanding the sample size and enriching the clinical dataset with information on non-motor symptoms of PD could enable a multifaceted classification of clinical subtypes. This approach would align with the framework proposed by Fereshtehnejad and colleagues, which incorporates non-motor features into the subtype categorization.^64^ Moreover, increasing the sample size would enhance the robustness of our results by enabling the detection of higher effect sizes across all factors considered in the statistical analysis, thus overcoming the limitations identified by the sensitivity analysis.

Secondly, while this study focused on happiness and fear, expanding the range of negative emotions that have also been shown to influence gait initiation would be of crucial importance. For instance, a recent study by Lebert and colleagues demonstrated that reaction times were shorter when healthy individuals observed angry facial expressions compared to fearful ones.^55^

On the other hand, results on how facial expressions depicting anger and sadness can modulate motor behaviour in PD are still scarce. A study by Ricciardi and colleagues^67^ showed that reaction times in recognizing anger and sadness were slower in PwPD compared to HC, but this was also associated with lower accuracy.^67^ Indeed, as shown by Lin *et al*.^62^ and, more extensively, in the reviews by Péron *et al*.^68^ and Argaud *et al*.,p^63^ PwPD have consistently shown a deficit in recognizing other negative emotions such as anger and sadness, with a higher deficit as the disease progresses towards a more severe state, but the impact of this aspect on motion and gait deserves to be explored.^62,63,68^ Hence, considering the use of other negative emotions in studies involving PwPD might give a more thorough insight into the effects of emotions in driving compensatory mechanisms to improve gait performance.

Finally, here we collected only behavioural data, but it would be of interest in future studies to explore the neural basis of motor compensation driven by emotional stimuli in PD, by using imaging techniques.

## Conclusions

To conclude, we demonstrated reduced compensatory mechanisms of motor performance triggered by ‘fear’ emotional stimuli with PD progression, but not in different motor subtypes. The motor advantage in response to ‘happiness’ stimuli, instead, differed between PD motor subtypes.

## Data Availability

The data supporting this study's findings are available from the corresponding author upon reasonable request.
